# The Psychedelic *N*,*N*-Dipropyltryptamine Prevents Seizures in a Mouse Model of Fragile X Syndrome via a Mechanism that Appears Independent of Serotonin and Sigma1 Receptors

**DOI:** 10.1021/acsptsci.3c00137

**Published:** 2023-09-18

**Authors:** Richa Tyagi, Tanishka S. Saraf, Clinton E. Canal

**Affiliations:** Department of Pharmaceutical Sciences, College of Pharmacy, Mercer University, 3001 Mercer University Drive, Atlanta, Georgia 30341, United States

**Keywords:** 5-HT_2A_, 5-HT_1B_, 5-HT_1A_, sigma1, psychedelics, fragile X syndrome, antiepileptic, polypharmacology

## Abstract

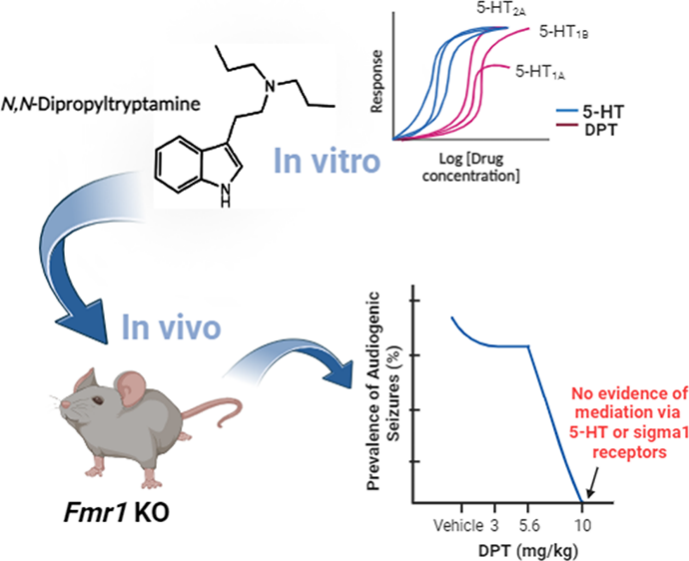

The serotonergic psychedelic psilocybin shows efficacy in treating neuropsychiatric disorders, though the mechanism(s) underlying its therapeutic effects remain unclear. We show that a similar psychedelic tryptamine, *N*,*N*-dipropyltryptamine (DPT), completely prevents audiogenic seizures (AGS) in an *Fmr1* knockout mouse model of fragile X syndrome at a 10 mg/kg dose but not at lower doses (3 or 5.6 mg/kg). Despite showing in vitro that DPT is a serotonin 5-HT_2A_, 5-HT_1B_, and 5-HT_1A_ receptor agonist (with that rank order of functional potency, determined with TRUPATH Gα/βγ biosensors), pretreatment with selective inhibitors of 5-HT_2A/2C_, 5-HT_1B_, or 5-HT_1A_ receptors did not block DPT’s antiepileptic effects; a pan-serotonin receptor antagonist was also ineffective. Because 5-HT_1A_ receptor activation blocks AGS in *Fmr1* knockout mice, we performed a dose–response experiment to evaluate DPT’s engagement of 5-HT_1A_ receptors in vivo. DPT elicited 5-HT_1A_-dependent effects only at doses greater than 10 mg/kg, further supporting that DPT’s antiepileptic effects were not 5-HT_1A_-mediated. We also observed that the selective sigma1 receptor antagonist, NE-100, did not impact DPT’s antiepileptic effects, suggesting DPT engagement of sigma1 receptors was not a crucial mechanism. Separately, we observed that DPT and NE-100 at high doses caused convulsions on their own that were qualitatively distinct from AGS. In conclusion, DPT dose-dependently blocked AGS in *Fmr1* knockout mice, but neither serotonin nor sigma1 receptor antagonists prevented this action. Thus, DPT might have neurotherapeutic effects independent of its serotonergic psychedelic properties. However, DPT also caused seizures at high doses, showing that DPT has complex dose-dependent in vivo polypharmacology.

Many studies are investigating the therapeutic potential of serotonergic psychedelics, including psilocybin and related psychedelic tryptamines, for various psychiatric conditions. Indications under study include but are not limited to major depressive disorder (MDD) and substance-use disorders, whereas other indications under consideration include autism spectrum disorder (ASD) and fragile X syndrome (FXS).^1−9^ Despite the proliferation of clinical studies, the specific pharmacodynamic properties that contribute to the therapeutic efficacies of psychedelics are not well understood. Psychedelic tryptamines are serotonin (5-HT) 5-HT_2A_, 5-HT_2B_, and 5-HT_2C_ receptor (5-HT_2_R) agonists, but they bind various other targets.^10−15^ For example, they are 5-HT_1A_ and 5-HT_1B_R agonists, and some, including *N,N*-dimethyltryptamine have direct modulatory effects in vivo on non-serotonergic receptors, including sigma1Rs.^11,16−18^ This poses the question of whether targets in addition to 5-HT_2_Rs contribute to the pharmacotherapeutic effects of psychedelics.^19,20^

We have been researching 5-HTRs as targets for treating FXS and ASD.^21−23^ FXS is a monogenic neurodevelopmental disorder that is the leading cause of intellectual disability and ASD.^24^ In addition to various other neurobehavioral issues, individuals with FXS present with auditory hypersensitivities and seizures.^25^ Seizures affect approximately 12% of FXS patients,^26−31^ with three times higher risk in comorbid ASD patients.^32^ 5-HT and its receptors were under investigation as novel antiepileptics decades ago, and there has been a resurgence of interest in this area, owing to the recently proven antiepileptic effects of the 5-HT releaser and low-potency, direct 5-HT_2_R agonist, fenfluramine, in Dravet syndrome and Lennox–Gastaut syndrome.^33−35^ Clinical trials are now underway assessing the efficacy of selective 5-HT_2C_R agonists for treating seizures in Dravet syndrome,^36^ based partly on historical studies showing susceptibility in 5-HT_2C_R knockout (KO) mice to audiogenic seizures (AGS).^37^ In some preclinical studies, nonselective activation of 5-HT_2_Rs attenuates generalized tonic–clonic and myoclonic seizures.^38−41^ A classical study showed that the serotonergic psychedelic 5-methoxy-*N,N*-dimethyltryptamine inhibited myoclonic seizures caused by photic stimulation in lateral geniculate-kindled felines.^42^ However, these observations are controversial, as some studies show no seizure-modulating effects of nonselective 5-HT_2_R activation.^43−45^ Still, others show that nonselective 5-HT_2_R *antagonists* block psychostimulant-induced convulsions,^46^ and there are clinical reports of seizures induced by certain, full-efficacy 5-HT_2A_ agonist psychedelics.^47^ Thus, the functions of distinct 5-HT_2_R subtypes in modulating distinct types of epilepsy remain unsolved.

With few exceptions, e.g., in absence seizures,^48,49^ in preclinical models, 5-HT_1A_R activation is antiepileptic. For example, it inhibits hippocampal focal seizures in felines and prevents AGS in the *Fmr1* KO mouse model of FXS.^50−52^ WAY-100635, a selective 5-HT_1A_R antagonist, inhibits the anti-AGS effect in *Fmr1* KO mice of the selective 5-HT_1A_ agonist, NLX-112.^50^ WAY-100635 also inhibits the anticonvulsant effects of the 5-HT_1A/1B_R agonist RU24969 on pentylenetetrazol-induced seizures and inhibits the anticonvulsant effects of the 5-HT_1A_R agonist 8-OH-DPAT on picrotoxin-induced seizures in mice,^45,50,53^ demonstrating that activation of 5-HT_1A_Rs is antiepileptic in various seizure models. Several other preclinical studies show that selective serotonin reuptake inhibitors (SSRIs) decrease the occurrence and increase the threshold of various types of induced seizures.^41,54−56^ This provides ample evidence that targeting the central 5-HT system may be a fruitful approach for treating epilepsies.

Sigma1Rs are another target of tryptamines that can modulate epileptiform activity.^11,57−59^ Also, fenfluramine was shown to act as a positive allosteric modulator at sigma1Rs in mice and zebrafish models,^60^ and sigma1R modulation prevents seizures in models of Dravet syndrome, amphetamine-induced seizures, and epileptic encephalopathies.^59,61,62^ We tested the hypothesis that the serotonergic psychedelic *N,N*-dipropyltryptamine (DPT) would prevent AGS in juvenile *Fmr1* KO mice and that it would be effective via a serotonergic or sigma1R mechanism. DPT is a short-acting psychedelic tryptamine, but there is limited knowledge about its pharmacology and behavioral effects; in vivo, it possesses agonist activity at 5-HT_1A_ and 5-HT_2A_Rs,^63−66^ and in vitro it has been shown to be a substrate of the 5-HT transporter.^14^ DPT is not restricted as a Schedule 1 controlled substance and hence is accessible for laboratory research without possessing a Drug Enforcement Agency controlled substances license. Here, we report observations that DPT prevents AGS in *Fmr1* KO mice, but our in vitro and in vivo pharmacology experiments did not provide evidence that its antiepileptic effects were 5-HTR- or sigma1R-mediated.

## Results

### DPT Is an Antiepileptic in *Fmr1* KO Mice

**Figure 1:**
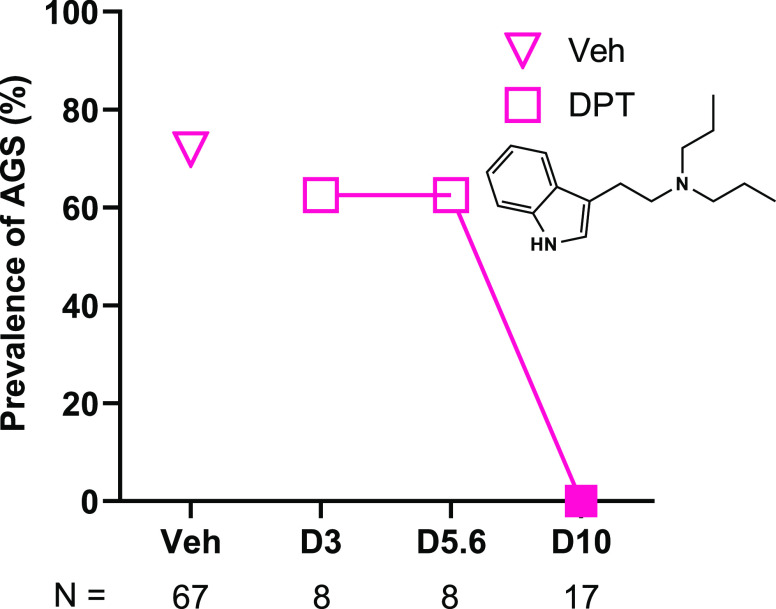
Dose-effect study of DPT (structure shown) on AGS in juvenile *Fmr1* KO mice. DPT at 10 mg/kg, but not 3 or 5.6 mg/kg prevented AGS. The filled square denotes the statistically significant effect of DPT compared to vehicle (Veh, *p* < 0.0001). D3, D5.6, D10: DPT 3, 5.6 and 10 mg/kg. *N* = number of subjects per group. The vehicle group includes new (*N* = 14) and historical (*N* = 53) data collected in our laboratory, to increase statistical power.^96^

Compounds that target the central 5-HT system, such as fenfluramine, have antiepileptic effects in individuals with neurodevelopmental disorders.^33,34,41,67^ Hence, we evaluated the antiepileptic effects of DPT in juvenile *Fmr1* KO mice using the AGS assay. As shown in Figure 1, vehicle-treated male and female *Fmr1* KO mice showed a prevalence of AGS of 72%, and DPT completely prevented AGS at 10 mg/kg (*p* < 0.0001). Sixty-five percent of mice treated with 10 mg/kg DPT showed normal behavior during the presentation of the seizure-eliciting alarm (akin to wild-type (WT) mice), whereas 35% showed a wild-running and jumping (WRJ) response. The duration of WRJ in these mice was significantly longer than WRJ in vehicle-treated mice (vehicle (mean ± SEM), 22 ± 6.26 s vs 10 mg/kg DPT (mean ± SEM), 78 ± 20.7 s; *p* < 0.01) which could indicate that DPT treatment prevented the transition to the tonic–clonic seizure (TCS) stage of AGS in these mice.^68,69^ Relative to vehicle, DPT did not significantly affect the prevalence of AGS at 3 (*p* = 0.69) or 5.6 mg/kg (*p* = 0.69). In addition, 3 and 5.6 mg/kg DPT did not impact latency to seizure onset, seizure duration, or lethality caused by AGS (*p* values ≥ 0.46, Supplemental Figure S1).

### In Vitro, DPT Is a Modest Potency 5-HT_2A_R Agonist, and In Vivo, DPT Elicits Peak 5-HT_2A_R-Dependent Head-Twitch Responses at a Dose Equivalent to Its Antiepileptic Dose

**Figure 2:**
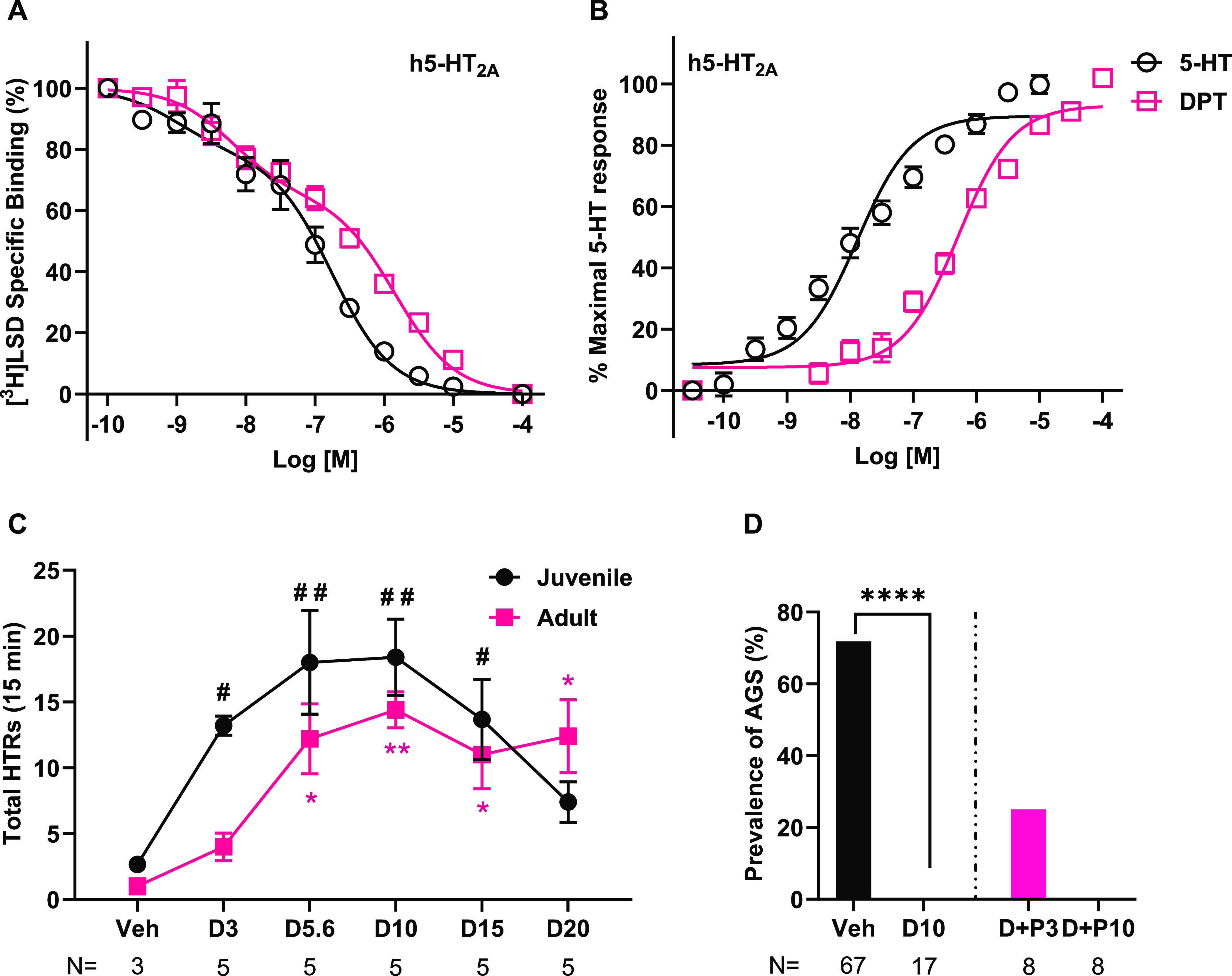
In vitro and in vivo pharmacology of DPT at 5-HT_2A_Rs and examination of the impact of inhibiting 5-HT_2A/2C_Rs on the antiepileptic properties of DPT. (A) In vitro radioligand competition binding of DPT and 5-HT at human (h) 5-HT_2A_Rs. The 100 μM data point was interpolated, so curves reached asymptote (no specific binding). Data were obtained from two separate experiments in which 5-HT was tested in duplicate and DPT was tested in sextuplicate per concentration. Data best fit to a “two-site, fit *K*_i_” model, which is shown. (B) In vitro, functional activity of 5-HT and DPT at h5-HT_2A_Rs. Data were obtained from three experiments in which 5-HT and DPT were tested in quadruplicate per concentration. (C) DPT dose-dependently elicited the 5-HT_2A_R-dependent HTR, with maximal effects at 10 mg/kg in juvenile and adult WT mice. (We previously found no difference in DOI-elicited HTRs between WT and *Fmr1* KO mice.^22^) Note that this dose was equivalent to the effective dose of DPT to prevent seizures, demonstrating that DPT engaged 5-HT_2A_Rs while it prevented seizures. *represents *p* < 0.05 and ^##^, **represents *p* < 0.01 compared to vehicle. Despite this, as shown in (D), the selective 5-HT_2A/2C_R antagonist, pimavanserin, did not significantly block the anti-AGS effects of DPT in juvenile *Fmr1* KO mice. **** represents *p* < 0.0001 DPT 10 mg/kg compared to vehicle; data reproduced from Figure 1 to show the comparison to the pimavanserin-treated groups. Veh: Vehicle; D3, 5.6, 10, 15 and 20: DPT 3, 5.6, 10, 15 and 20 mg/kg; P3 and 10: pimavanserin 3 and 10 mg/kg. *N* = number of mice tested. All data with error bars are means and SEMs.

We next tested DPT’s in vitro and in vivo pharmacology at 5-HT_2A_Rs to explore whether 5-HT_2A_R activation could be mediating its antiepileptic effects. In vitro, DPT was a moderate potency full agonist at 5-HT_2A_Rs with *E*_max_ of 106% ± 1.13 (mean ± SEM), relative to 5-HT, the positive control. EC_50_ and *K_i_* values are reported in Table 1. See Figure 2A,B for affinity and function nonlinear regression curves.

**Table 1:** DPT and 5-HT Affinities (*K*_i_) and EC_50_ Values, Determined Using TRUPATH Gαβγ Biosensors, at Human 5-HT_2A_, 5-HT_1A_, and 5-HT_1B_Rs Expressed in HEK293T Cells^a^

	5-HT	DPT
	5-HT_2A_	
*K*_i_ high, nM	0.5	3
p*K*_i_ high (95% CI)	9.29 (10.23 to 8.47)	8.49 (8.81 to 8.18)
*K*_i_ low, nM	90	739
p*K*_i_ low (95% CI)	7.04 (7.23 to 6.78)	6.13 (6.29 to 5.95)
EC_50_, nM	12	451
pEC_50_ (95% CI)	7.92 (8.08 to 7.76)	6.35 (6.48 to 6.22)
	5-HT_1A_	
*K*_i_ , nM	26	1641
p*K*_i_ (95% CI)	8.49 (8.81 to 8.18)	5.78 (5.87 to 5.70)
EC_50_, nM	69	2430
pEC_50_ (95% CI)	7.16 (7.21 to 7.12)	5.61 (5.71 to 5.52)
	5-HT_1B_	
*K*_i_, nM	15	8081
p*K*_i_ (95% CI)	7.81 (7.89 to 7.74)	5.09 (5.14 to 5.04)
EC_50_, nM	0.4	1210
pEC_50_ (95% CI)	9.39 (9.56 to 9.23)	5.92 (6.15 to 5.69)

a [^3^H]LSD was used to label 5-HT_2A_Rs; [^3^H]5-CT was used to label 5-HT_1A_ and 5-HT_1B_Rs. For 5-HT_2A_Rs, the affinity data fit best to a “two–site–fit *K*_i_” model. *K*_i_s and p*K*_i_s values are means. CI = confidence interval.

In vivo, DPT produced a dose-dependent head-twitch response (HTR), with maximal effects at 10 mg/kg (Figure 2C). In juvenile WT mice (P23–25), we observed a main effect of treatment (*F*(5, 23) = 4.25, *p* = 0.007). Relative to vehicle treatment, DPT increased the number of HTRs at 3, 5.6, 10, and 15 mg/kg doses (mean ± SEM HTRs vehicle = 3 ± 0.3; DPT 3 mg/kg = 13 ± 0.7, *p* = 0.047; DPT 5.6 mg/kg = 18 ± 3.9, *p* = 0.007; DPT 10 mg/kg = 18 ± 2.9, *p* = 0.007; DPT 15 mg/kg = 14 ± 3.1, *p* = 0.046). HTRs decreased at the 20 mg/kg dose, generating the classic bi-phasic dose-effect curve (mean ± SEM HTRs elicited by DPT 20 mg/kg = 7 ± 1.5, *p* = 0.287, compared to vehicle).

In adult WT mice (∼2 months old), we observed a similar main effect of treatment (*F*(5, 22) = 5.18, *p* = 0.027). Relative to vehicle, 3 mg/kg DPT was not sufficient to elicit the HTR (mean ± SEM HTRs vehicle = 1 ± 0.6 and DPT 3 mg/kg = 4 ± 1.0, *p* = 0.391). DPT elicited a significant HTR at all other doses tested, i.e., 5.6, 10, 15, and 20 mg/kg (mean ± SEM HTRs DPT 5.6 mg/kg = 12 ± 2.7, *p* = 0.013; DPT 10 mg/kg = 14 ± 1.4, *p* = 0.004; DPT 15 mg/kg = 11 ± 2.6, *p* = 0.016; DPT 20 mg/kg = 12 ± 2.8, *p* = 0.013, compared to vehicle). (±)-2,5-Dimethoxy-4-iodoamphetamine (DOI) at 1 mg/kg—used as a positive control—elicited a very high number of HTRs in both juvenile (3 vehicle vs 35 DOI, *p* = 0.001) and adult (1 vehicle vs 28 DOI, *p* < 0.001) WT mice, thus validating our assay (Supplemental Figure S2). Additionally, DPT at all five test doses did not affect locomotor activity in adult WT mice, but in juvenile WT mice, at 20 mg/kg, DPT reduced the distance traveled (Supplemental Figure S3). The dose of DPT that elicited the highest number of HTRs (10 mg/kg) was the DPT dose that effectively blocked AGS, suggesting 5-HT_2A_R activation might mediate DPT’s antiepileptic effects.

### Antagonism of 5-HT_2A/2C_Rs Does Not Block DPT’s Antiepileptic Effects

As DPT engaged 5-HT_2A_Rs in vivo at 10 mg/kg, which matched its effective dose to block AGS, we tested whether selective antagonism of 5-HT_2A/2C_Rs blocks DPT’s anti-AGS effects (Figure 2D). Pretreatment with 3 mg/kg pimavanserin slightly reversed the antiepileptic effects of DPT 10 mg/kg (AGS prevalence 25% for pimavanserin 3 mg/kg plus DPT 10 mg/kg vs AGS prevalence of 0% for DPT 10 mg/kg alone), but this increase in the seizure prevalence was not significant (*p* = 0.10). Pretreatment with pimavanserin 10 mg/kg did not impact DPT’s anti-AGS effects (AGS prevalence 0 vs 0%; *p* > 0.99). Mice treated with pimavanserin 3 mg/kg behaved normally, i.e., like vehicle-treated mice. Mice treated with pimavanserin 10 mg/kg showed signs of mild sedation which included partial ptosis, low sensory responses, hypolocomotion, and immobility that took effect within 2 min of injection and which lasted ∼30 min. These observations suggest that the anti-AGS effects of DPT are not mediated by 5-HT_2A_/_2C_R activation. These data align with our previous observations that the 5-HT_2C_R-preferring agonist, lorcaserin, does not prevent AGS in juvenile *Fmr1* KO mice.^70^

### In Vitro, DPT Is a Low-Potency 5-HT_1A_R Agonist, and In Vivo, DPT Elicits 5-HT_1A_R-Dependent Behavioral Effects at Doses Higher than Its Antiepileptic Dose

**Figure 3:**
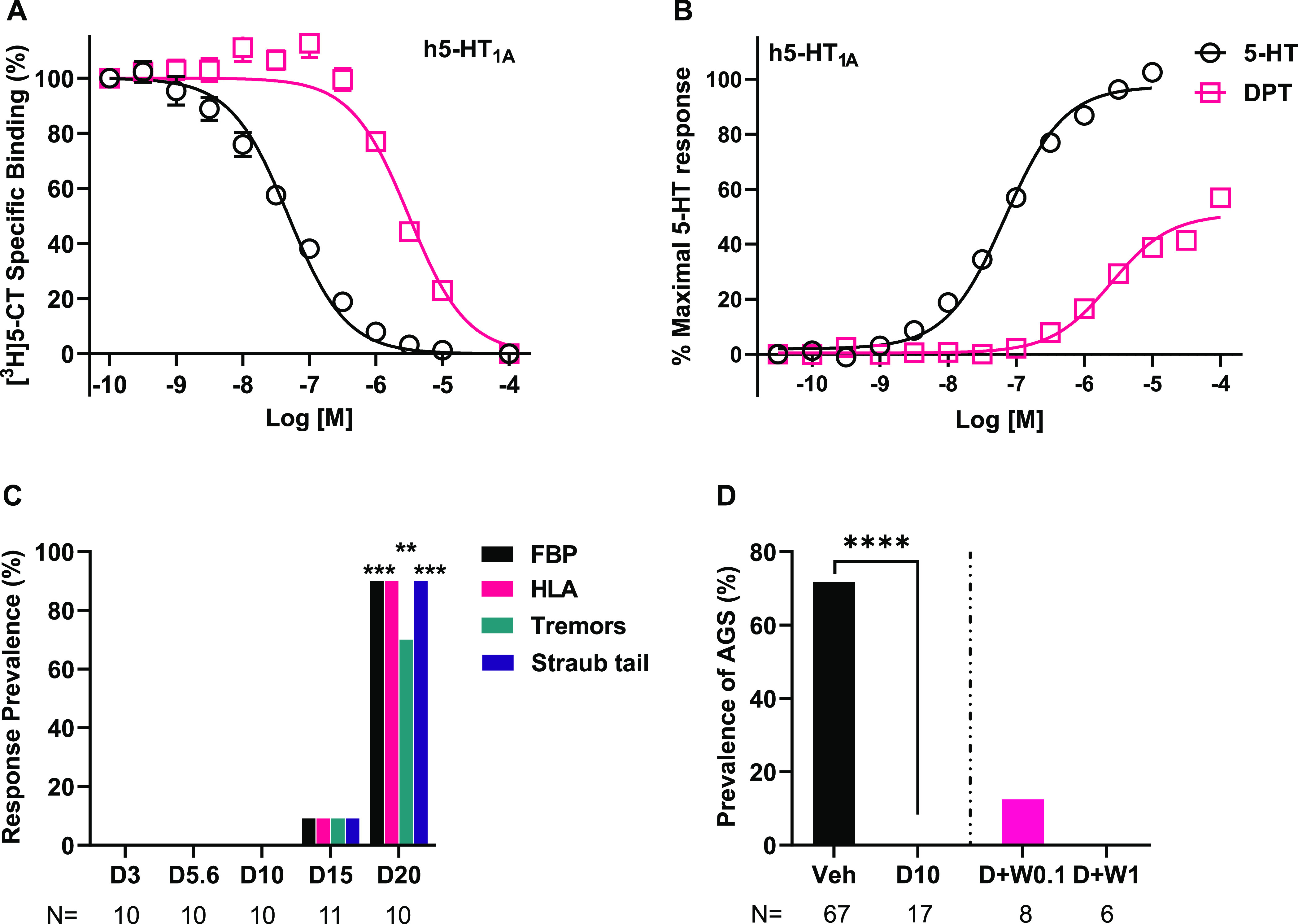
In vitro and in vivo pharmacology of DPT at 5-HT_1A_Rs and examination of the impact of inhibiting 5-HT_1A_Rs on the antiepileptic effects of DPT. (A) In vitro radioligand competition binding of DPT and 5-HT at human (h) 5-HT_1A_Rs. The 100 μM data point was interpolated, so curves reached asymptote (no specific binding). Data were obtained from two separate experiments in which 5-HT was tested in duplicate and DPT was tested in sextuplicate per concentration. (B) In vitro functional activity of 5-HT and DPT at h5-HT_1A_Rs. Data were obtained from four experiments in which 5-HT and DPT were tested in quadruplicate per concentration. (C) DPT at 20 mg/kg elicited behavioral signs of 5-HT_1A_R engagement in juvenile and adult WT mice including flat body posture (FBP), hind limb abduction (HLA), tremors, and Straub tail; DPT did not elicit these behaviors at its antiepileptic dose of 10 mg/kg. **, *** represents *p* < 0.01 and *p* < 0.001, respectively, compared to vehicle. (D) The selective 5-HT_1A_R antagonist, WAY-100635, did not block the antiepileptic effects of DPT in juvenile *Fmr1* KO mice. **** represents *p* < 0.0001 DPT 10 mg/kg compared to vehicle; data reproduced from Figure 1 to show the comparison to the WAY-100635-treated groups. Veh: Vehicle; D3, 5.6, 10, 15 and 20: DPT (3, 5.6, 10, 15 and 20 mg/kg); W0.1 and W1: WAY-100635 (0.1 and 1 mg/kg). *N* = Number of mice tested. All data with error bars are means and SEMs.

As we and others recently showed that 5-HT_1A_R activation blocks AGS in *Fmr1* KO mice,^50,51^ we next tested whether DPT activation of 5-HT_1A_Rs could be mediating its anti-AGS effects. We tested its pharmacology at 5-HT_1A_Rs in vitro and in vivo. In vitro, we observed that DPT is a low potency partial agonist at 5-HT_1A_Rs, with an *E*_max_ of 53% ± 3.67 (mean ± SEM) relative to 5-HT, the positive control. These observations are similar to a previous study of DPT at 5-HT_1A_Rs.^71^ EC_50_ and *K_i_* values are reported in Table 1. See Figure 3A and 3B for affinity and function curves. As shown in Figure 3C, relative to vehicle, juvenile and adult mice treated with DPT at 20 mg/kg, but not lower doses, showed distinct behavioral symptoms, which included flat body posture (0 vs 90%, *p* = 0.0001), hind limb abduction (0 vs 90%, *p* = 0.0001), tremors (0 vs 70%, *p* = 0.003), and Straub tail (0 vs 90%, *p* = 0.0001). These behaviors are 5-HT_1A_R-dependent,^50,72^ providing evidence that DPT does not sufficiently engage 5-HT_1A_R at its antiepileptic dose of 10 mg/kg.

### Antagonism of 5-HT_1A_Rs Does Not Block DPT’s Antiepileptic Effects

To further evaluate whether DPT’s antiepileptic effects in the AGS model were 5-HT_1A_R-dependent, we tested whether selective antagonism of 5-HT_1A_Rs blocks DPT’s effects. Pretreatment with WAY-100635 0.1 and 1 mg/kg did not significantly reverse DPT’s anti-AGS effects (13% vs 0% *p* = 0.33, and 0% vs 0% *p* > 0.99, respectively) (Figure 3D). Importantly, we previously showed that WAY-100635 at a 0.1 mg/kg dose blocks the antiepileptic effects of the highly selective 5-HT_1A_R agonist, NLX-112, in the AGS assay in *Fmr1* KO mice.^50^ Thus, these data support the conclusion that DPT’s antiepileptic effects are not 5-HT_1A_R-mediated.

### In Vitro, DPT Is a Very Low-Potency 5-HT_1B_R Agonist, and In Vivo, Antagonism of 5-HT_1B_ Receptors Does Not Block DPT’s Antiepileptic Effects

**Figure 4:**
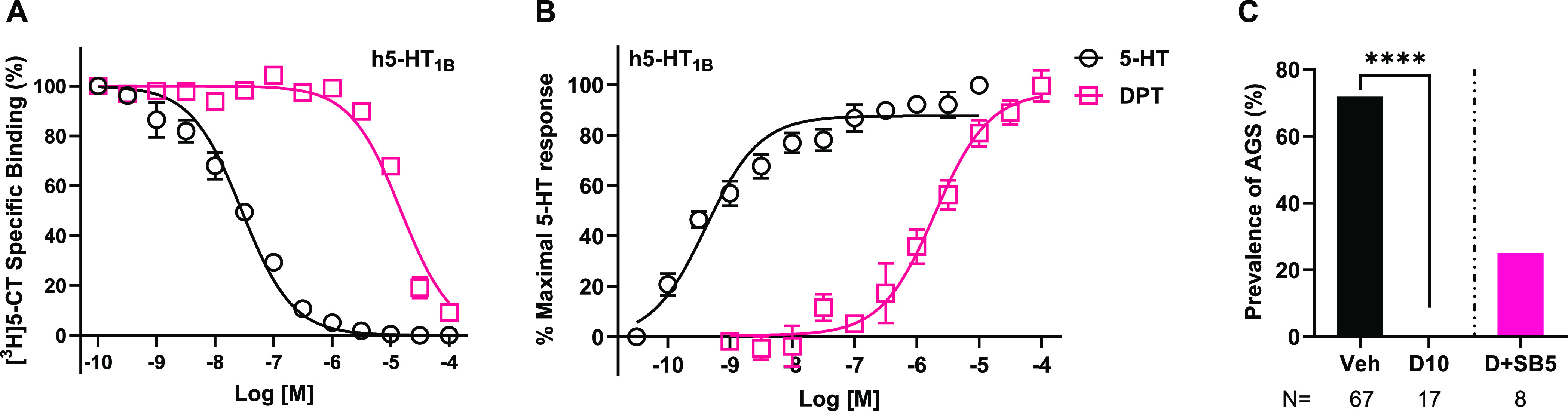
In vitro and in vivo pharmacology of DPT at 5-HT_1B_Rs and examination of the impact of inhibiting 5-HT_1B_Rs on the antiepileptic properties of DPT. (A) In vitro, radioligand competition binding of DPT and 5-HT at human (h) 5-HT_1B_Rs. The 100 μM data point was interpolated, so curves reached asymptote (no specific binding). Data were obtained from four separate experiments in which 5-HT was tested in duplicate and DPT was tested in sextuplicate per concentration. (B) In vitro functional activity of 5-HT and DPT at h5-HT_1B_Rs. Data were obtained from four individual experiments per assay in which 5-HT and DPT were tested in quadruplicate per concentration. (C) The selective 5-HT_1B_R antagonist, SB-224289, did not block the antiepileptic effects of DPT in juvenile *Fmr1* KO mice. **** represents *p* < 0.0001 DPT 10 mg/kg compared to vehicle; data reproduced from Figure 1 to show the comparison to the SB-224289-treated group. Veh: Vehicle; D10: DPT 10 mg/kg; SB5: SB-224289 5 mg/kg. *N* = number of mice tested. All data with error bars are means and SEMs.

We next tested DPT’s in vitro pharmacology at 5-HT_1B_Rs and observed that it is a very low-potency 5-HT_1B_R agonist, relative to 5-HT, the positive control. DPT’s *E*_max_ was 83% ± 1.95 (mean ± SEM), relative to 5-HT. EC_50_ and *K_i_* values are reported in Table 1. See Figure 4A,B for affinity and function curves. We tested whether selective 5-HT_1B_R antagonism would block DPT’s anti-AGS effects. As shown in Figure 4C, pretreatment with SB-224289 at 5 mg/kg increased seizure prevalence when compared to treatment with DPT 10 mg/kg alone (25 vs 0%). However, the difference was not statistically significant (*p* = 0.10). These observations suggest that activation of 5-HT_1B_Rs does not mediate DPT’s anti-AGS effects.

### Pan-5-HTR Antagonism Does Not Block DPT’s Antiepileptic Effects

**Figure 5:**
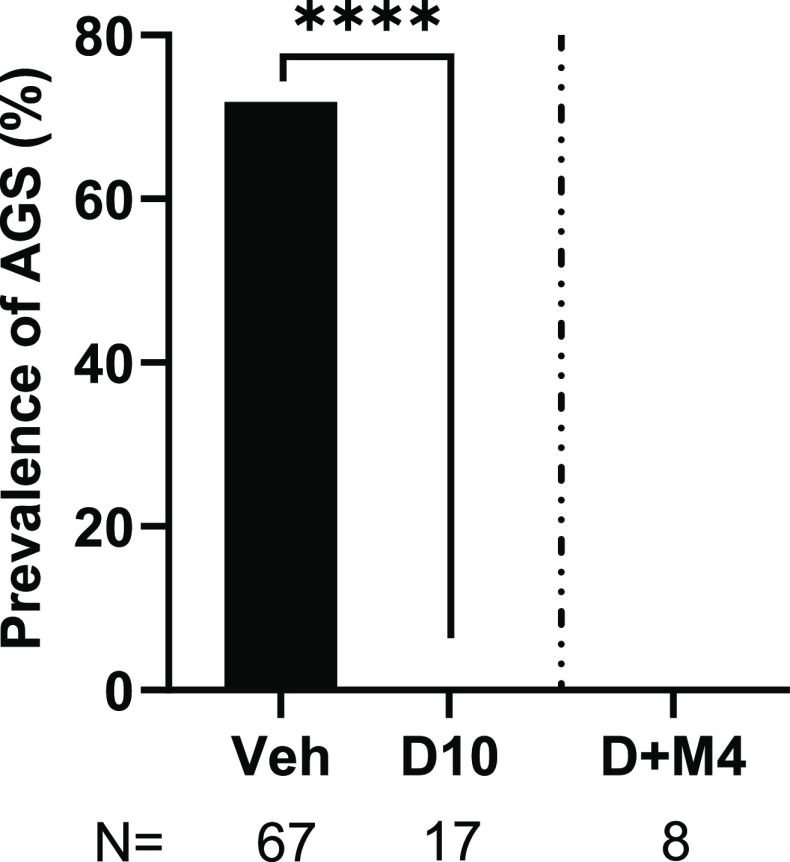
The pan-5-HTR inhibitor methiothepin did not block the anti-AGS effects of DPT in juvenile *Fmr1* KO mice. **** represents *p* < 0.0001 DPT 10 mg/kg compared to vehicle; data reproduced from Figure 1 to show the comparison to the methiothepin-treated group. Veh: Vehicle; D10: DPT 10 mg/kg; M4: Methiothepin 4 mg/kg. *N* = number of mice tested.

Given DPT’s structural similarity and shared targets with 5-HT, and our objective to investigate the potential involvement of multiple 5-HTRs in mediating the anti-AGS effects of DPT, we next tested the effects of the pan-5-HTR antagonist methiothepin. Pretreatment with methiothepin at 4 mg/kg did not influence DPT’s anti-AGS effects (seizure prevalence 0 vs 0%; *p* > 0.99) (Figure 5). During the observation period, mice pretreated with methiothepin appeared to be highly sedated (partial or complete ptosis) while lying in a prone or medial-lateral position being either slightly responsive or unresponsive to the AGS-eliciting alarm. These effects were more prominent in the methiothepin-treated group as compared to the pimavanserin 10 mg/kg group (data not shown). We did a pilot test of pretreatment with methiothepin at 2 mg/kg; it also did not impact the antiepileptic effects of DPT (data not shown). Collectively, our observations suggest that the anti-AGS effects of DPT may be mediated by a mechanism(s) that is independent of 5-HTRs.

### Antagonism of Sigma1Rs Does Not Block DPT’s Antiepileptic Effects

**Figure 6:**
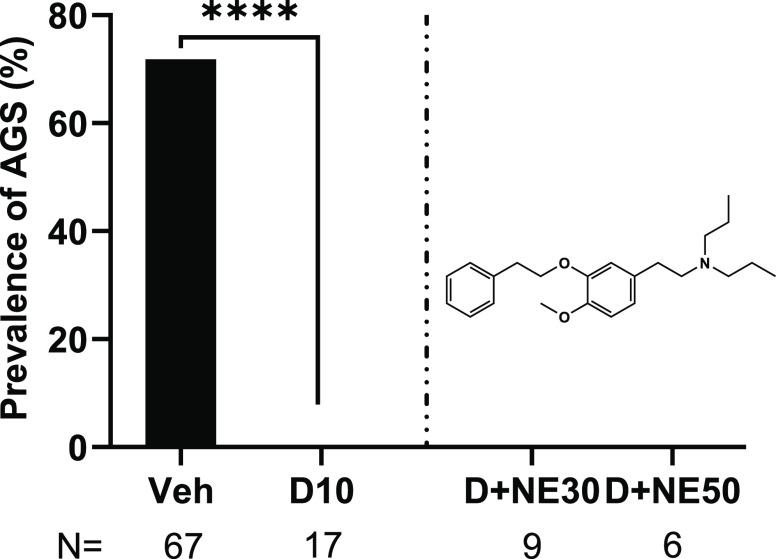
Examination of the impact of sigma1R blockade on the antiepileptic effects of DPT in juvenile *Fmr1* KO mice. NE-100 (structure shown to illustrate the shared *N,N*-dipropyl moiety in NE-100 and DPT) at a subconvulsive dose (30 mg/kg) and at a convulsive dose (50 mg/kg) did not influence the anti-AGS effect of DPT. Note that this figure reports AGS and not NE-100-elicited seizures; see Table 2 for descriptive differences in the types of seizures. **** represents *p* < 0.0001 DPT 10 mg/kg compared to the vehicle; data reproduced from Figure 1 to show the comparison to the NE-100-treated groups. Veh: Vehicle; D10: DPT 10 mg/kg; NE30 and NE50: NE-100 30 and 50 mg/kg, respectively. *N* = number of mice tested.

Since no 5-HTR antagonist challenged DPT’s antiepileptic efficacy in the AGS assay, we tested whether a nonserotonergic receptor could mediate DPT’s antiepileptic effects. DPT and other tryptamines bind sigma1Rs, which studies have shown modulate epileptic activity.^14,59,61,62,73^ NE-100, a selective sigma1R antagonist, is structurally similar to DPT, sharing an *N,N*-dipropyl moiety^74^ (see Figures 1 and 6). NE-100 potentiates seizures at a 25 mg/kg dose and induces seizures at 50 mg/kg and above.^75^ To determine suitable doses of NE-100 for evaluation in the AGS assay—doses of NE-100 that straddle the threshold dose for NE-100 causing seizures on its own—we tested NE-100 at six different doses, 5, 10, 15, 20, 30, and 50 mg/kg, in juvenile WT and *Fmr1* KO mice, and observed them for 30 min following administration. Like a previous report,^75^ we observed that 50 mg/kg NE-100 induced generalized TCS (Table 2), whereas the mice exhibited normal behavior when administered the lower doses. Thus, we tested the impact of 30 mg/kg and 50 mg/kg NE-100 on DPT’s anti-AGS effects. Neither 30 mg/kg nor 50 mg/kg NE-100 pretreatment reversed the anti-AGS effects of 10 mg/kg DPT (all mice showed no AGS; 0%, *p* > 0.99) (Figure 6).

**Table 2:** AGS, 20 mg/kg DPT and 50 mg/kg NE-100 Elicited Seizures Are Qualitatively Distinct^a^

responses	AGS	DPT seizures	NE-100 seizures
early	startle response	tail lifting	flat body with an abdominal stretch
	squinting of eyes	loss of balance	tail lifting or whipping
	wild running and jumping	tremors	loss of balance and trembling
	brief opisthotonus		brief oral movements
during	clonus, lying on the side	clonus with repeated falling	clonus, lying on the back or side with repeated falling
	tonic extension of limbs and tail	tonic convulsion while sitting with prominent opisthotonos (≈10 s) with quick recovery (2–3 s)	tonic extension of limbs with continuous, alternating kicking and uncoordinated movements of limbs followed by continuous rolling on the floor
	duration: ∼13 s	duration: ∼15 s	duration: ∼150 s
late	∼71% lethality; ∼29% quickly recovered, accompanied by brief wild running and jumping	0% lethality. Presence of hind-limb abduction, flat body posture	0% lethality. ∼20–30 min to recover alertness and controlled movements

a Descriptions of AGS were gathered from experiments with vehicle-treated juvenile *Fmr1* KO mice. Descriptions of seizures elicited by 20 mg/kg DPT were gathered from the dose–effect HTR study of DPT using juvenile and adult WT mice. Descriptions of seizures elicited by 50 mg/kg NE-100 were gathered from a dose-determining study of NE-100 using juvenile WT and *Fmr1* KO mice and tests of NE-100 in the AGS study using juvenile *Fmr1* KO mice.

### DPT and NE-100 Cause Seizures on Their Own that Are Qualitatively Distinct from AGS

During dose–response testing of DPT and NE-100, we observed that at high doses, i.e., 20 mg/kg DPT and 50 mg/kg NE-100, both compounds caused seizures on their own. Hence, we compared the behavioral signs of convulsions due to AGS, DPT, and NE-100; each caused a unique time-dependent repertoire of behavioral symptoms (Table 2). DPT 20 mg/kg induced convulsions in 90% of mice tested (*N* = 10 total WT mice tested). These began between 5 and 10 min after administration, and the duration was brief. Mice exhibited myoclonic tremors that transitioned to tonic convulsions with prominent opisthotonos, and 50% of the mice vocalized during the episode. The entire seizure episode lasted less than 20 s. All mice recovered, and then showed behavioral signs of 5-HT_1A_R activation (see Table 2). One adult male mouse also showed excessive salivation after the seizure.

NE-100 50 mg/kg induced convulsions in 69% of mice tested (*N* = 13 total tested, including 10 *Fmr1* KO and 3 WT mice). Behaviors that preceded seizures and behaviors indicative of seizures were similar to those reported by Vavers et al.^75^ NE-100-induced seizures lasted 150–200 s, and mice recovered to normal behavior between 20 and 30 min after treatment. Like DPT, NE-100-induced seizures were not lethal. Importantly, we did not observe a treatment by genotype effect (Supplemental Figure S4). In the AGS experiments, co-treatment with NE-100 50 mg/kg and DPT 10 mg/kg (*N* = 8, the same subjects as those in Figure 6) caused seizures within ∼2 min of administration, i.e., prior to sounding the AGS-eliciting alarm. These seizures presented the same symptomology as observed with NE-100 50 mg/kg alone. After sounding the alarm, the characteristics of the seizures observed were like NE-100-induced seizures. In other words, mice did not exhibit AGS (see Methods and Table 2). In conclusion, DPT and NE-100 caused seizures that were qualitatively distinct from AGS, suggesting different mechanisms.

## Discussion

We discovered that DPT prevents AGS in juvenile *Fmr1* KO mice, a genetic model of FXS. We also report DPT’s affinity and function at 5-HT_2A_, 5-HT_1A_, and 5-HT_1B_ receptors. DPT was effective at preventing AGS only at a 10 mg/kg dose, whereas at lower doses AGS persisted. In separate studies of in vivo receptor engagement, DPT activated 5-HT_2A_ receptors, i.e., elicited the HTR,^76,77^ at its antiepileptic dose. It took a higher dose to engage 5-HT_1A_Rs, e.g., to elicit hind-limb abduction and flat body posture, which are elicited by 5-HT_1A_R activation.^72,78^ These results corroborated our in vitro data which showed DPT was a moderate-potency full agonist at 5-HT_2A_Rs but a low-potency partial agonist at 5-HT_1A_Rs. We also showed that DPT was a low-potency 5-HT_1B_R agonist in vitro. The in vitro and in vivo receptor pharmacology studies suggested that DPT engaged 5-HT_2A_Rs but not 5-HT_1A_ or 5-HT_1B_Rs at its antiepileptic dose. As an additional approach to investigate if these receptors contributed to DPT’s antiepileptic properties, we tested if co-administration of selective 5-HT_2A/2C_, 5-HT_1A_, or 5-HT_1B_R antagonists could block DPT’s antiepileptic effects. None did nor did a pan 5-HTR antagonist. Collectively, our results suggest that DPT blocks AGS in juvenile *Fmr1* KO mice via a nonserotonergic mechanism.

Interestingly, we observed that at high doses, DPT switched from an antiepileptic (in the AGS assay) to a proconvulsant, eliciting tonic seizures when administered on its own. The proconvulsant effects of DPT that we observed at 20 mg/kg align with a study in rats that showed DPT was proconvulsant at 30 mg/kg.^64^ Such dose-dependent switches in effects were also described with the 5-HT_1_R agonist, sumatriptan. Sumatriptan increases pentylenetetrazol-induced seizure thresholds in mice at 1 mg/kg but reduces the threshold at 20 mg/kg, suggesting the engagement of different targets or neural circuits at different doses.^79^ Similarly, 5-methoxy-*N,N*-dimethyltryptamine, mentioned in the Introduction section as having antiseizure effects in lateral geniculate kindled felines, has also been reported anecdotally to induce convulsions—akin to seizures—in humans when administered at strong doses (see experience ID:39420 and 76059 at Erowid.org). Based on the available evidence, the effects of DPT are dose-dependent, consistent with DPT having polypharmacology like other tryptamines.

Sigma1Rs are targets of several tryptamines^11^ and modulate epileptiform activity,^59^ which provided us the rationale to investigate them as antiepileptic targets of DPT. We used the sigma1R antagonist, NE-100, based on its structural similarity to DPT. Two observations lead us to conclude that DPT’s anti-AGS effects were not caused by activation *or* inactivation of sigma1Rs. NE-100 failed to reverse DPT’s effects, and DPT failed to impact (either suppress or potentiate) NE-100-elicited seizures. Also, NE-100 caused characteristically distinct convulsions at a 50 mg/kg dose. These seizures differed from AGS and DPT-induced seizures in terms of the behavioral sequelae and duration. Furthermore, drug-elicited seizures differ in underlying anatomical loci than AGS. Drug-elicited seizures affect various neural systems,^80^ whereas in *Fmr1* KO mice, AGS have a localized origin, being dependent on altered activity in the inferior colliculus, an auditory pathway structure in the midbrain.^81^

One possible mechanism for why DPT was antiepileptic in the AGS assay is that it directly modulates auditory processing, reducing auditory hypersensitivity in *Fmr1* KO mice. In humans, a closely related tryptamine *N,N*-diisopropyltryptamine reduces sound pitch and causes harmonic distortion while keeping the relationship between tones intact; subjects report that sounds from music are an octave lower than usual, i.e., as if they are listening to music underwater.^63^ The possibility that DPT or related tryptamines can target auditory processing is worth exploring in the future, as it may help improve understanding of auditory hypersensitivity in FXS and other neurodevelopmental disorders.

A limitation of the in vivo pharmacological antagonism studies is the side-effect of sedation (no locomotion, flaccid bodies, and eyes closed or partially closed) caused by pimavanserin and methiothepin. The sedation caused by brain-wide inhibition of 5-HT_2A/2C_Rs in the case of pimavanserin and brain-wide inhibition of 5-HT_1_, 5-HT_2_, 5-HT_3_, 5-HT_5_, 5-HT_6_, 5-HT_7_Rs (and other receptors) in the case of methiothepin may have been sufficient to block auditory signals from reaching the inferior colliculus to cause AGS. For example, 5-HT_2A_R blockade in the frontal cortex may have diminished auditory processing, and potentiated the anti-AGS effects of DPT, i.e., could have had anti-AGS effects independent of DPT. However, we previously showed that the selective 5-HT_2A_R antagonist/inverse agonist, M100907, which causes sedation in mice, does not block AGS in juvenile *Fmr1* KO mice.^70^ Another possibility for the inefficacy of pimavanserin to block the anti-AGS effects of DPT is that DPT’s effects were due to precise, localized modulation of 5-HT_2A_Rs. 5-HT_2A_R modulation—5-HT_2A_R biased signaling, antagonism or agonism of distinct 5-HT_2A_R signal transduction pathways—in auditory neural pathways might have underlied the antiepileptic effects of DPT, and we were unable to detect this because of brain-wide inhibition of 5-HT_2A_Rs that obfuscated this effect. It is yet to be determined whether local blockade or inactivation of 5-HT_2A_Rs in auditory neural pathways would block DPT’s anti-AGS effects, and conversely, whether local activation of 5-HT_2A_Rs by DPT would be sufficient to block AGS.

DPT has not been studied extensively. A PubMed search of articles with “dipropyltryptamine” in their abstracts produced only 23 results. Little is known about DPT’s pharmacodynamics. We investigated DPT’s functional effects at 5-HT_2A_, 5-HT_1A_, and 5-HT_1B_Rs, using new TRUPATH technology, which probes the activity of ligands to stimulate individual Gα subunits coupled to GPCRs, and often, ligands have unique potencies to activate different Gα subunits.^82,83^ Future studies might find, for example, that DPT has different potencies at 5-HT_1A_ and 5-HT_1B_ coupled to Gα_i/o_ family subunits other than Gα_i3_, which we examined. One study assessed DPT’s affinity and function at 5-HT_1A_ receptors, using [^3^H]8-OH-DPAT and GTPγS incorporation, respectively;^71^ DPT’s 5-HT_1A_R affinity was substantially higher than the affinity we measured with [^3^H]5-CT competition binding, but it was a 5-HT_1A_R partial agonist, like we observed. Another study reported that DPT was inactive at 5-HT_1A_Rs up to 10 μM but used calcium mobilization as the functional readout.^12^ The higher affinity of DPT at 5-HT_1A_ compared to 5-HT_1B_ that we observed is similar to other psychedelic tryptamines.^11^ DPT’s functional activity at 5-HT_2A_Rs was also measured by Blough et al.,^12^ and its potency to stimulate canonical 5-HT_2A_-Gα_q_ signaling was higher than what we observed with TRUPATH; still, its full agonist efficacy was consistent with our results. Finally, there is no information to our knowledge about DPT’s pharmacokinetics in any species. Thus, we are parsimonious in our conclusion about DPT’s in vivo mechanism(s).

We conjecture that our observations gel with recent research that concludes that some effects of psychedelics are mediated by nonserotonergic mechanisms.^19,20^ Our observations of an apparent nonserotonergic mechanism underlying the antiepileptic effects of DPT add to the growing literature about the pharmacological mechanisms underlying the potential therapeutic effects of serotonergic psychedelics.

## Methods

### Animals

All experimental protocols involving FVB.129P2-*Pde6b^+^ Tyr^c-ch^ Fmr1^tm1Cgr^/J* (*Fmr1* KO mice, stock #004624, Jackson Laboratory) and FVB.129P2-*Pde6b^+^ Tyr^c-ch^/AntJ* (sighted FVB or WT mice, stock #004828) were approved by the Mercer University Institutional Animal Care and Use Committee and were performed following the Guide for the Care and Use of Laboratory Animals, 8th edition. We used *Fmr1* KO juvenile mice (P23–P25), male and female, for tests of AGS. The mice were bred and raised in the vivarium at Mercer University College of Pharmacy as previously described.^22^ All tests were performed during the light cycle (7:00–19:00).

### Compounds

DPT hydrochloride, NE-100 hydrochloride, DOI hydrochloride, and methiothepin maleate were purchased from Cayman Chemical. WAY-100635 maleate was purchased from Tocris. Pimavanserin was obtained from Selleckchem, and SB-224289 hydrochloride was purchased from R&D Systems. 5-HT hydrochloride and mianserin hydrochloride were obtained from Alfa Aesar. For in vivo pharmacology tests, all compounds were dissolved in Milli-Q (Millipore Sigma) water, which served as the vehicle, except for SB-224289, which was dissolved in 2% DMSO, 4% Tween-80, and 4% PEG-20 and subsequently q.s. with Milli-Q. The solutions were made fresh on the day of the experiments. Vehicle and all compounds for in vivo studies were administered intraperitoneally (i.p.) to *Fmr1* KO and WT mice at a volume of 1 mL/100 g. Doses of compounds were selected based on studies showing their in vivo efficacy. For in vitro pharmacology studies, 10 mM stocks of test ligands were prepared in DMSO. [^3^H]Lysergic acid diethylamide (LSD) and [^3^H]5-Carboxamidotryptamine (5-CT) were purchased from PerkinElmer and were diluted in assay buffer.

### Cell Growth, Maintenance, and Transfection

Plasmids encoding human 5-HT_2A_, 5-HT_1A_, and 5-HT_1B_Rs were obtained from the cDNA Resource Center. Dulbecco’s modified Eagle’s medium (DMEM) and OptiMEM were obtained from Gibco. Fetal bovine serum (FBS) and dialyzed FBS (dFBS) were purchased from Corning Life Sciences and Gibco. HEK 293 T cells (CRL-3216, ATCC) were used for in vitro binding and functional assays. Cells were cultured in 10 cm dishes with DMEM medium containing 10% FBS and were maintained in an incubator at 37 °C, 5% CO_2_, and 95% humidity.

For radioligand competition binding assays, cells were transfected at ∼80% confluency with 7–10 μg of cDNA and 40 μg of transfection grade polyethyleneimine (PEI, 40,000 molecular weight, Polysciences, Inc., prepared as 1 mg/mL in Milli-Q). The transfection cocktail was prepared by separately mixing PEI and plasmids in two vials containing 2.5 mL of OptiMEM and then subsequently combining them. After incubating the transfection cocktail for 30 min at 37 °C, cells were washed with phosphate-buffered saline then cells were gently covered with the transfection cocktail together with 5 mL DMEM and a final concentration of 5% dFBS (transfection media). For the TRUPATH functional assays, cells at ∼80% confluency were transfected with 5-HT_2A_, 5-HT_1A_, and 5-HT_1B_R cDNA (5–10 μg), Rluc8-Gα_q_ for 5-HT_2A_ and Rluc8-Gα_i3_ for 5-HT_1A_ and 5-HT_1B_Rs, untagged Gβ_3_, and GFP2-Gγ_9_ plasmids in 1:1:1 ratio (750 ng) using the same transfection protocol described earlier.

### Radioligand Competition Binding

Cell membranes expressing 5-HT_2A_, 5-HT_1A_, and 5-HT_1B_Rs were collected after 48 h of transfection. Cells were collected and homogenized in ice-cold 50 mM Tris HCl buffer. Homogenate was spun thrice at 12,000 × *g* for 10 min at 4 °C using an Avanti JXN-26 centrifuge (Beckman Coulter). The supernatant was discarded after each spin and the final pellet was stored at −80 °C for later testing. Competition binding assays with DPT and control compounds were performed in 96 well plates, using ∼0.7 nM [^3^H]LSD to radiolabel 5-HT_2A_Rs, and ∼0.2 and ∼0.3 nM [^3^H]5-CT to radiolabel 5-HT_1A_ and 5HT_1B_Rs, respectively. Nonspecific binding was determined in the presence of 10 μM mianserin for 5-HT_2A_Rs, 10 μM serotonin for 5-HT_1A_Rs, and 10 μM SB-224289 for 5-HT_1B_Rs. After the addition of assay buffer (50 mM tris–HCl, 10 mM MgCl_2_, and 0.1 mM EDTA, pH = 7.4 at room temperature), test ligands, radioligand, and cell membranes expressing 5-HT_2A_, 5-HT_1A_, or 5-HT_1B_ Rs, the plates were covered and incubated on a shaker for 90 min at room temperature. Plate contents were rapidly filtered through Whatman GF/B filter mats using a 96-well cell harvester (PerkinElmer) and then washed with ∼800 mL ice-cold 50 mM Tris·HCl to remove unbound radioligand. Filter mats were dried and saturated with a scintillation cocktail (ScintiVerse Cocktail, Fisher Scientific), and scintillations were counted using a PerkinElmer Microbeta 2 instrument.

### Bioluminescence Resonance Energy Transfer 2 Assay (TRUPATH)

Cells were plated in white opaque 96-well microplates (Perkin Elmer) 48 h after transfection in bioluminescence resonance energy transfer (BRET) buffer at a density of 50,000 cells/well. After 2 h in an incubator, cells were treated with freshly prepared luminescent enzyme substrate coelenterazine (5 μM). After 5 min of the equilibration period, 5-HT (positive control) and DPT were added to the wells. After another 5 min, plates were then read in an LB940 Mithras plate reader (Berthold Technologies, Oak Ridge, TN) with 395 nm (RLuc8-coelenterazine 400a) and 510 nm (GFP2) emission filters. G-protein activation was measured as BRET2 ratios (the ratio of the GFP2 emission to RLuc8 emission).^83^

### Audiogenic Seizures

Experiments testing the induction of AGS in *Fmr1* KO were conducted as previously described.^22^ Juvenile mice were acclimated to the test room for 30–60 min in their home cages. Mice were then administered vehicle or DPT at 3, 5.6, or 10 mg/kg. To determine the potential contribution of 5-HT_2A_, 5-HT_1A,_ 5-HT_1B_, other 5-HTRs, and sigma1Rs to the antiepileptic effects of DPT, separate groups of mice were pretreated with the selective 5-HT_2A_R antagonist/inverse agonist pimavanserin (3 and 10 mg/kg),^84^ the selective 5-HT_1A_R antagonist WAY100635 (0.1 and 1 mg/kg),^85,86^ the selective 5-HT_1B_R antagonist SB-224289 (5 mg/kg),^87^ the pan-5-HTR antagonist methiothepin (2 and 4 mg/kg),^88,89^ and the sigma1R antagonist NE-100 (30 and 50 mg/kg)^75^ 10 min before treatment with DPT (10 mg/kg). All mice were placed back in their cages and were tested 5 min after injection with DPT. Pretreatment periods were decided based on prior studies conducted in rodents.^90−93^ Also, we previously showed that 0.1 mg/kg WAY100635 at this pretreatment interval is effective at preventing the anticonvulsant effects of the selective 5-HT_1A_R agonist, NLX-112.^50^ Mice were placed in a clear, polycarbonate box (46 cm × 20 cm × 20 cm) covered with a perforated, clear, polycarbonate lid 1 min before being exposed to an alarm (RadioShack Kit #49-1010, doorstop alarm). The alarm was held by hand ∼10 cm away from the test box and the duration of exposure was 5 min. A sound-level meter/data logger (REED Model SD-4023) was placed ∼20 cm from the alarm and read during testing to ensure a uniform level of sound pressure in each experiment. Tests were video-recorded using a high-definition camcorder (Vixia HF R800, Canon). A maximum of 4 mice (2 per box) were observed simultaneously by two experimenters.^70^ The average (±standard deviation (SD)) baseline sound pressure in the testing room was 55 ± 9 dB, and the average alarm sound pressure was 105 ± 4 dB.

Behavioral responses, including normal behavior, WRJ, TCS, and death, were documented during AGS testing. Normal behavior was defined as coordinated locomotion, alertness, exploring, sniffing, sitting, rearing, grooming, socializing, and squinting of eyes. The beginning of AGS was marked by a startle response, squinting of eyes, followed by WRJ phase(s), brief opisthotonos, a clonic phase with the mouse lying on either side of its body with head, neck, trunk, and limbs ventro-flexed (muscle jerking and twitching with rigidity), a short (∼5 s) tonic seizure phase with full extension of extremities (muscle stiffening), and finally, respiratory arrest. Seizure was defined by TCS. In the case of recovery from the TCS phase, mice exhibited a second round of WRJ, Straub tail, a full body vibrating shudder, and tremors which finally ended with either freezing or a transition to normal behavior. The frequencies of AGS were documented by visual observations of video recordings.^70^

### DPT 5-HT_2A_R and 5-HT_1A_R In Vivo Pharmacology

WT mice were acclimated to a procedure room for ≥30 min before administering test compounds. Juvenile (P23-P25) and adult (>P60) mice were injected (i.p.) with Milli-Q water (vehicle) or DPT (3, 5.6, 10, 15, or 20 mg/kg) and were immediately placed in a clear open-field plexiglass chamber (43 × 43 cm; Med Associates). 5-HT_2A_R-dependent HTRs were counted using a hand-held tally counter for 15 min postinjection. Locomotor activity (distance traveled in cm) was video recorded and calculated by Ethovision software (Noldus Information Technology). Observations of 5-HT_1A_R-dependent effects (see Figure 3C) were also documented.

### Statistical Analysis

Statistical tests were performed using GraphPad Prism, version 9. AGS and other behaviors were analyzed using Fisher’s exact test (two-sided, α = 0.05). To evaluate the efficacy of various doses of DPT to elicit the HTR compared to vehicle, a one-way ANOVA with Holm-Šídák’s multiple comparisons test was used. Student’s *t* test was used for HTR comparison between DOI and vehicle treatment. Nonlinear regression was used for analyzing in vitro pharmacology results. Of note, for the 5-HT_2A_R binding, data fit best to a two-site model. For 5-HT and DPT binding at 5-HT_1A_R and 5-HT_1B_R, we used a one-site model.^94,95^*K*_d_ values were set to 0.78 nM for [^3^H]LSD at 5-HT_2A_Rs, and 0.2 and 0.3 nM for [^3^H]5-CT at 5-HT_1A_ and 5-HT_1B_Rs, respectively, and were based on values reported in the literature.

## Supporting Information Available

Figure S1, latency to and duration of WRJ and TCS in *Fmr1* KO mice in the AGS assay; Figure S2, effect of 1 mg/kg DOI on the HTR in juvenile and adult WT mice; Figure S3, effect of 3, 5.6, 10, 15, and 20 mg/kg DPT on locomotor activity in WT mice; Figure S4, latency to and duration of seizures induced by 50 mg/kg NE-100 in juvenile WT and *Fmr1* KO mice (PDF)
