# Supplementary material for: The Psychedelic N,N-Dipropyltryptamine Prevents Seizures in a Mouse Model of Fragile X Syndrome via a Mechanism that Appears Independent of Serotonin and Sigma1 Receptors
Source: ACS Pharmacol Transl Sci. 2023 Sep 18;6(10):1480–91. doi: 10.1021/acsptsci.3c00137 (PMC10580393; doi:10.1021/acsptsci.3c00137)
Supplement: Supplementary file 1 — pt3c00137_si_001.pdf [file pt3c00137_si_001.pdf]

<u>Supporting information</u>

The Psychedelic *N*,*N*-Dipropyltryptamine Prevents Seizures in a Mouse Model of Fragile X Syndrome via a Mechanism that Appears Independent of Serotonin and Sigma1 Receptors

Richa Tyagi[1], Tanishka S. Saraf[1], Clinton E. Canal[1*]

[1]Mercer University
College of Pharmacy
Department of Pharmaceutical Sciences
3001 Mercer University Drive
Atlanta, GA 30341

*Email: canal_ce@mercer.edu
Phone: (678) 547-6252

Supplemental figures:

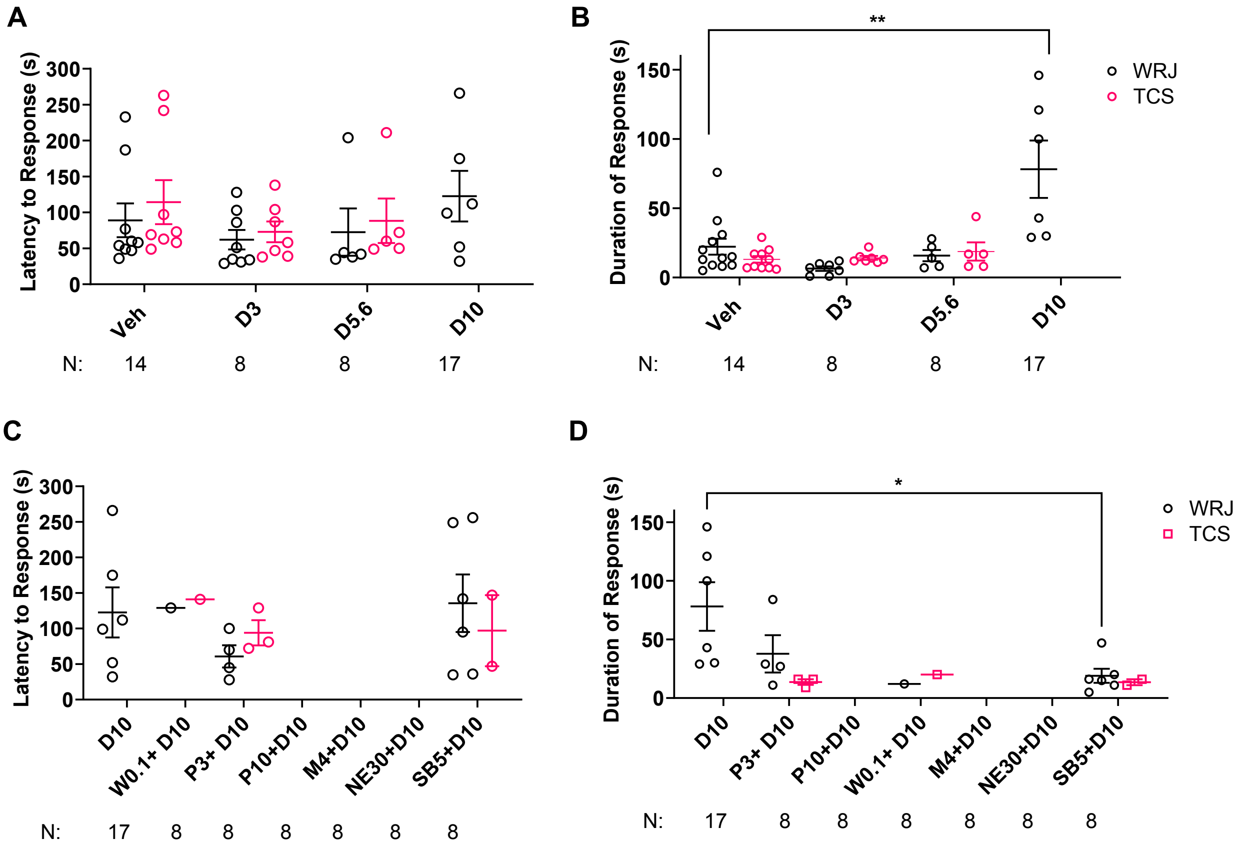

Figure S1. Effect of DPT alone or in combination with 5-HTR and sigma1R antagonists on latency to and duration of WRJ and TCS in *Fmr1* KO mice in the AGS assay. (A) DPT did not impact WRJ or TCS latencies. (B) DPT 10 mg/kg increased the duration of WRJ compared to vehicle treated subjects. **represents p<0.01 relative to vehicle. (C) Pimavanserin 3 and 10 mg/kg, WAY-100635 0.1 mg/kg, SB-224289 5 mg/kg, NE-100 30 mg/kg, and methiothepin 4 mg/kg treatment did not decrease the latency to WRJ or TCS compared to mice treated with DPT 10 mg/kg. (D) SB-224289 decreased the duration of WRJ in 25% of mice that had seizures. *represents p<0.05 relative to DPT 10 mg/kg. Veh: Vehicle; D3, 5.6, and 10: DPT 3, 5.6, and 10 mg/kg; P3 and 10: Pimavanserin 3 and 10 mg/kg; W0.1: WAY-100635 0.1 mg/kg; M4: Methiothepin 4 mg/kg; NE30: NE-100 30 mg/kg; SB5: SB-224289 5 mg/kg. N= number of mice tested. Data were analyzed with one-way ANOVAs with Dunnet’s post-hoc test. Data are scatter plots with means and SEMs indicated.

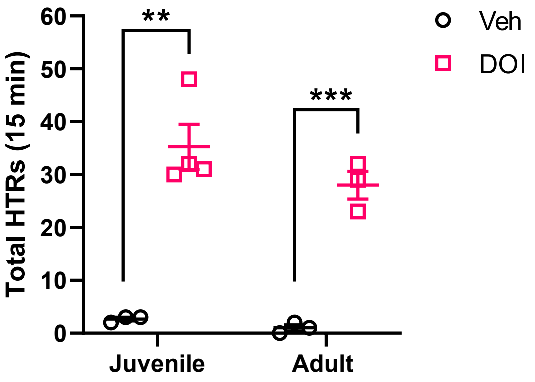

Figure S2. Effect of 2,5-Dimethoxy-4-iodoamphetamine (DOI) on the head twitch response (HTR) in WT mice. DOI elicited significantly higher HTRs than the vehicle in juvenile and adult WT mice. **represents p<0.01 and *** represents p<0.001 relative to vehicle. Data were analyzed with Student's t-tests. Data are scatter plots with means and SEMs indicated.

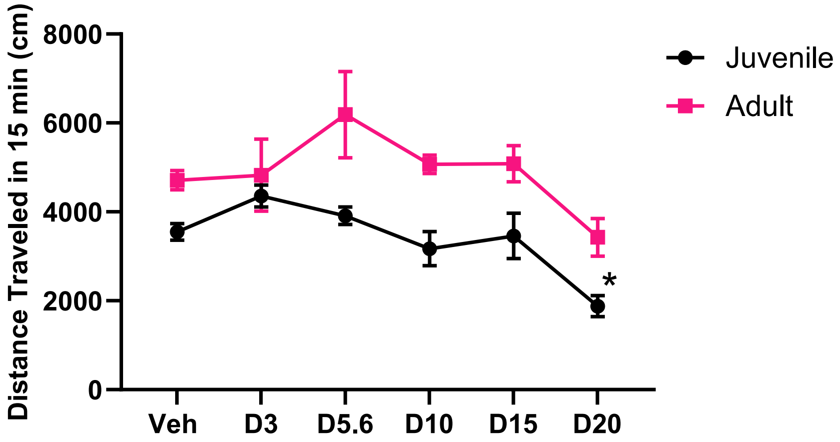

Figure S3. Effect of DPT on locomotor activity in WT mice. Relative to vehicle, DPT at 3, 5.6, 10, 15 and 20 mg/kg had no effect on distance traveled (0-15 min) in adult WT mice. DPT at 20 mg/kg decreased locomotion in juvenile WT mice. *represents p<0.5 relative to vehicle. Locomotor data were collected simultaneously with HTR data shown in Figure 2C of the main text. Veh: Vehicle; D3, 5.6, 10, 15 and 20: DPT 3, 5.6, 10, 15 and 20 mg/kg. Data were analyzed by a one-way ANOVA with Holm-Šídák's multiple comparisons test. Data are means and SEMs.

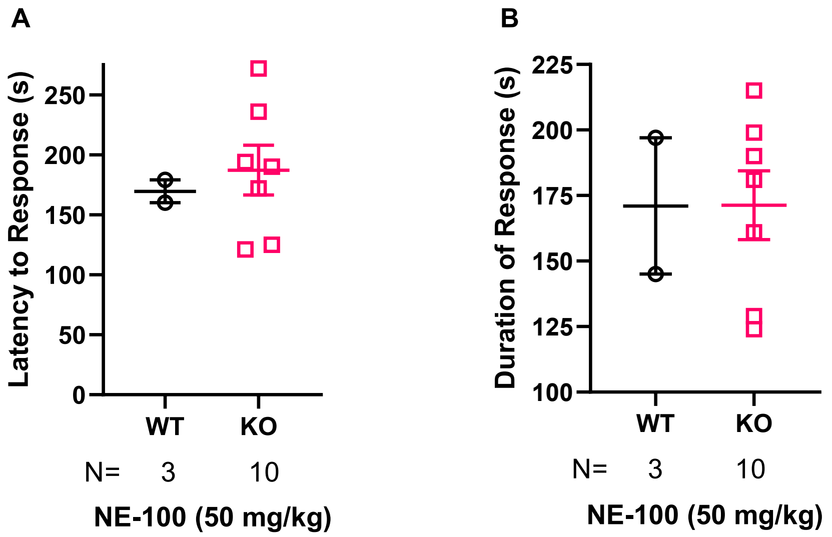

Figure S4. Latency to (A) and duration of (B) NE-100 induced seizures in juvenile WT and *Fmr1* KO mice. There were no differences between WT and *Fmr1* KO mice. Data were analyzed by Student t-tests. N= Total number of mice tested; some subjects did not show a response, hence the discrepancies in N's and data points shown in the figures. Data are scatter plots with means and SEMs indicated.
